# The invasive mosquito *Aedes japonicus japonicus* is spreading in northeastern Italy

**DOI:** 10.1186/s13071-019-3387-x

**Published:** 2019-03-26

**Authors:** Fabrizio Montarsi, Simone Martini, Alice Michelutti, Graziana Da Rold, Matteo Mazzucato, Davide Qualizza, Domenico Di Gennaro, Marcella Di Fant, Marco Dal Pont, Manlio Palei, Gioia Capelli

**Affiliations:** 1 Istituto Zooprofilattico Sperimentale delle Venezie, Legnaro, Italy; 2 Entostudio s.r.l, Ponte San Nicolò, PD Italy; 3 Azienda per l’Assistenza Sanitaria A.A.S. 3-Alto Friuli, Collinare e Medio Friuli, S.O.C. Igiene e Sanità Pubblica, Gemona del Friuli, Udine, Italy; 4 Azienda Sanitaria Universitaria Integrata di Udine-Dipartimento di Prevenzione A.S.S. 4-Medio Friuli, Udine, Italy; 5 Regione Autonoma Friuli Venezia Giulia, Direzione Centrale Salute, Integrazione Sociosanitaria e Politiche Sociali-Servizio Sanità Pubblica Veterinaria, Trieste, Italy

**Keywords:** *Aedes j. japonicus*, Invasive mosquito species, Entomological surveillance, Italy

## Abstract

**Background:**

The invasive mosquito species, *Aedes japonicus japonicus*, was detected in northeastern Italy for the first time in 2015, at the border with Austria. After this finding, a more intensive monitoring was carried out to assess its distribution and to collect biological data. Herein, we report the results of four years (2015–2018) of activity.

**Methods:**

The presence of *Ae. j. japonicus* was checked in all possible breeding sites through collections of larvae. The monitoring started from the site of the first detection at the Austrian border and then was extended in all directions. The mosquitoes were identified morphologically and molecularly.

**Results:**

*Aedes j. japonicus* was found in 58 out of 73 municipalities monitored (79.5%). In total (2015–2018), 238 sampling sites were monitored and 90 were positive for presence of *Ae. j. japonicus* larvae (37.8%). The mosquito was collected mainly in artificial containers located in small villages and in rural areas. Cohabitation with other mosquito species was observed in 55.6% of the samplings.

**Conclusions:**

*Aedes j. japonicus* is well established in Italy and in only four years has colonised two Italian Regions, displaying rapid spreading throughout hilly and mountainous areas. Colonization towards the south seems limited by climatic conditions and the occurrence of a large population of the larval competitor, *Ae. albopictus*. The further spread of *Ae. j. japonicus* has the potential to pose new threats of zoonotic agents (i.e. *Dirofilaria* spp. and West Nile virus) within areas at altitudes previously considered at negligible risk in Italy.

**Electronic supplementary material:**

The online version of this article (10.1186/s13071-019-3387-x) contains supplementary material, which is available to authorized users.

## Background

The Asian bush or rock pool mosquito, *Aedes* (*Finlaya*) *japonicus japonicus* (Theobald, 1901) (syn. *Hulecoeteomyia japonica*) (Diptera: Culicidae), is one of the most invasive mosquito species (IMS) worldwide and has spread throughout North America and Europe. In its native area of East Asia (Japan, Korea, southern China, southeastern Russia), *Ae. j. japonicus* occurs in temperate regions [1]. Currently, *Ae. j. japonicus* is reported from nine European countries, i.e. Belgium [2], the Netherlands [3], Switzerland [4], Germany [5], Austria, Slovenia [6], Hungary [7] and Croatia [8].

*Aedes j. japonicus* is not considered to be a major vector of pathogens in its native area, but its possible role as a vector of disease agents in other parts of the world is unclear; indeed, it seems able to transmit pathogens like flaviviruses and heartworms in laboratory studies [9, 10].

**Fig. 1:**
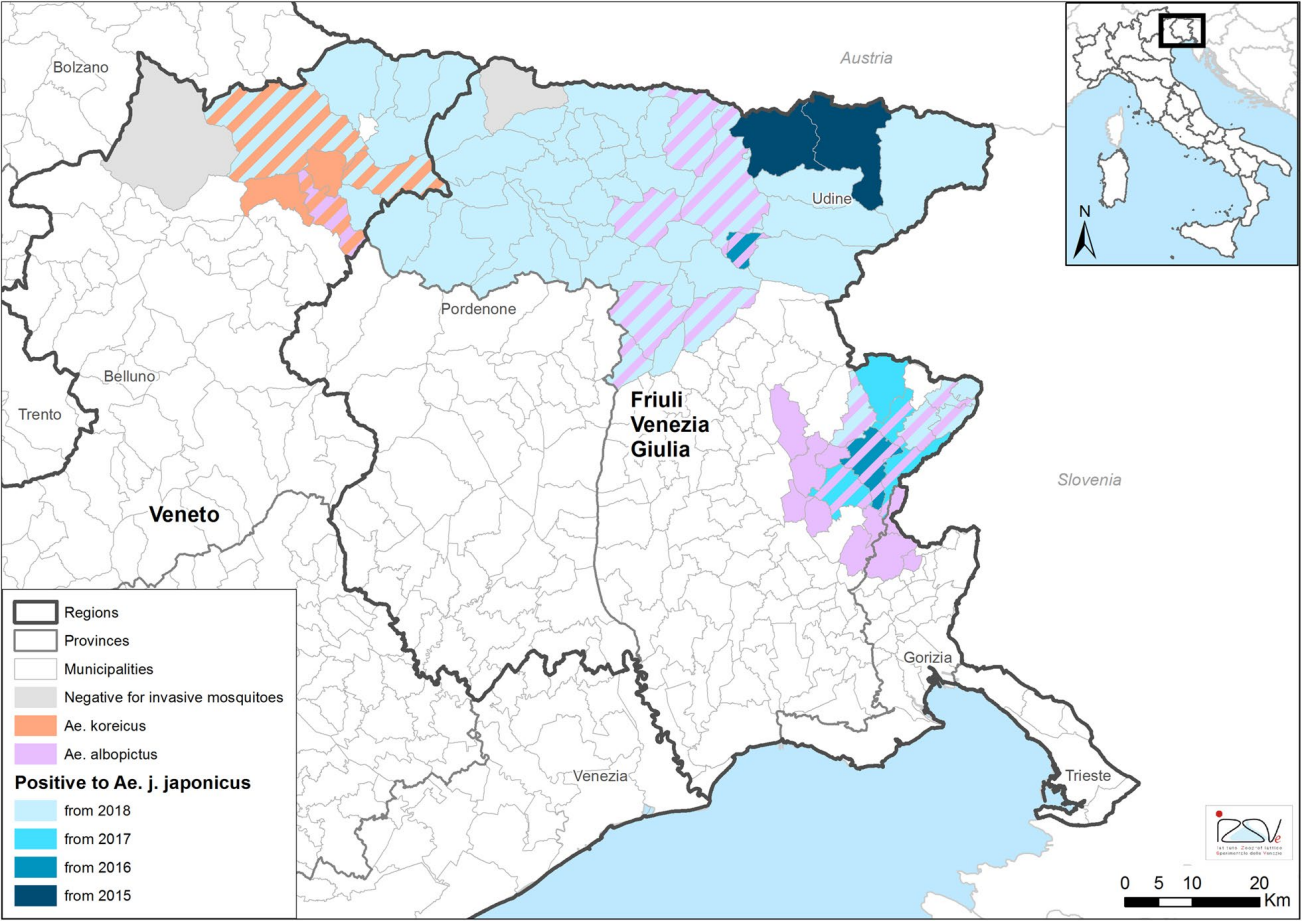
Map of the municipalities monitored and positive for the presence of *Aedes j. japonicus* in northeastern Italy, 2015–2018. Co-occurrence with *Aedes albopictus* and *Ae. koreicus* is also reported

A previous monitoring performed in Austria from 2011 to 2015 detected the mosquito in a village 25 km from the Italian border on July 2015. The researchers hypothesized that the species could spread to Italy and a following survey confirmed the presence of *Ae. j. japonicus* in three villages along the River Fella, Friuli Venezia Giulia (FVG) Region [11]. In October 2016, another unexpected finding occurred during a local survey in a village located in another area to south, close to the Slovenian border [Cividale del Friuli, 46°04′23.7″N, 13°26′00.4″E, 127 m above sea level (masl)] (Fig. 1).

In this part of northeastern Italy, the occurrence of two other IMS, *Aedes albopictus* (Skuse, 1895) (syn. *Stegomyia albopicta*) and *Aedes koreicus* (Edwards, 1917) (syn. *Hulecoeteomyia koreica*) was known [12]. Consequently, *Ae. j. japonicus* is the third Asian mosquito species occurring in Italy.

After the first findings, a more intensive monitoring was carried out to assess the current spreading. In this paper, we report the results of four years of monitoring on the occurrence and spread of *Ae. j. japonicus* in Italy. In addition, ecological data, such as breeding sites preference and coexistence with other mosquito larvae are also reported.

## Methods

### Study area

The area monitored is characterized by hills, mountains and valleys typical of the Dolomite Alps with an average elevation of 527.7 masl. The area has a sub-continental climate, characterized by a mild climate, with cold and snowy winters and mild warm summers. The average mean daily temperature ranges between 17–22 °C in the summer and between -2–3 °C in the winter. The annual rainfall is above 1000 mm. The human population density is low compared to other Italian areas (108.4 and 56 inhabitants/km^2^ in Udine and Belluno Provinces, respectively), and the inhabitants live mainly in small villages; only four have more than 10,000 inhabitants.

### Mosquito sampling and identification

At the time of the first findings of *Ae. j. japonicus* in the FVG Region there was no specific monitoring for invasive mosquitoes, which was activated only after the first report. Conversely, in Belluno Province a monitoring for IMS has been ongoing since 2011. The survey in FVG started in September 2015 near the most western site where the first *Ae. j. japonicus* mosquitoes were found (Pontebba; 46°30′16.9740″N, 13°18′10.8324″E; 561 masl). The monitoring was extended towards the west in 2016, following the Dolomites and their valleys with samplings in March, July and October. Since no further expansion in the area bordering Austria was found, in 2017 the monitoring focused on the area of the second finding (October 2016) bordering Slovenia, with samplings in June, July and September. In 2018, the surveillance was intensified throughout north FVG and in the bordering area of Veneto Region (Belluno Province) performing monthly samplings from April to November.

The detection of IMS should be performed with different methods and traps; however, due to budget and personnel constraints, we decided to focus the survey on the larval stage. Surveillance of larvae is considered one of the best methods in terms of targeted and rapid IMS detection and optimal for the cost-benefit ratio [13]. It is also well known that IMS develop mainly in artificial breeding containers; therefore, the surveillance of presence and availability of breeding sites was focused mostly at human settlements.

Larval collection was made using a standard larval dipper (500 ml, 10 cm diameter), checking all potential breeding sites present in each site, i.e. artificial containers, catch basins, tires and natural mosquito larval habitats. All the collection sites were georeferenced. The areas monitored included private and public places. When *Ae. j. japonicus* was found in an area, the surrounding environment was explored up to places with no longer positive for the species. Several sites negative for the presence of the species in 2015 were checked again in 2016 and 2018 as well as some positive sites to confirm its occurrence. The extention of the area colonized by *Ae. j. japonicus* was then estimated adding the surface of the municipalities where *Aedes japonicus* was recorded.

The larvae collected were identified morphologically as described in Montarsi et al. [14]. In case of the detection of *Ae. j. japonicus* for the first time in a municipality, at least one larval stage and eventually adults reared in the laboratory were confirmed through molecular biology. DNA was amplified using an in-house real-time SYBR green PCR, targeting two mitochondrial loci, nicotinamide adenine dinucleotide dehydrogenase subunit 4 gene (*nad*4, 480 bp) [15] and cytochrome *c* oxidase subunit 1 gene (*cox*1, 590/600 bp) [16], and one nuclear locus, *β tubulin* gene (BTUB, 370 bp) [17]. Briefly, the reactions were carried out in a total volume of 20 μl, containing, 5.8 μl of RNase free water, 10 μl of QuantiFast SYBR Green PCR Master Mix 2× (Qiagen GmbH, Hilden, Germany), 0.3 μM of sense and reverse primer and 3 μl of extracted DNA. Amplifications were performed in a StepOnePlus™ instrument (Applied Biosystems, Foster City, CA, USA). The thermal profile consisted of 5 min at 95 °C, followed by 40 cycles at 95 °C for 15 s, 55 °C for 30 s (for *nad*4 and BUTB primers), 58 °C for 30 s (for *cox*1 primers) and 60 °C for 30 s. Following amplification, dissociation was performed by slowly raising the temperature of the thermal chamber from 60 to 95 °C. Negative and positive controls were included in each run.

The amplicons were sequenced, and the sequences obtained compared with GenBank entries. Representative sequences were submitted to GenBank.

### Statistical analysis

The differences of *Ae. j. japonicus* prevalence (only place/breeding sites monitored more than 10 times) according to the municipality of collection and type of larval breeding sites were tested using the Chi-square test or Fisher’s exact test when appropriate using the free software WinEpi [18].

Maps were created using the GIS software ESRI® ArcMap™ version 10.5.1 offered by ArcGIS™ Desktop [19].

## Results

**Fig. 2:**
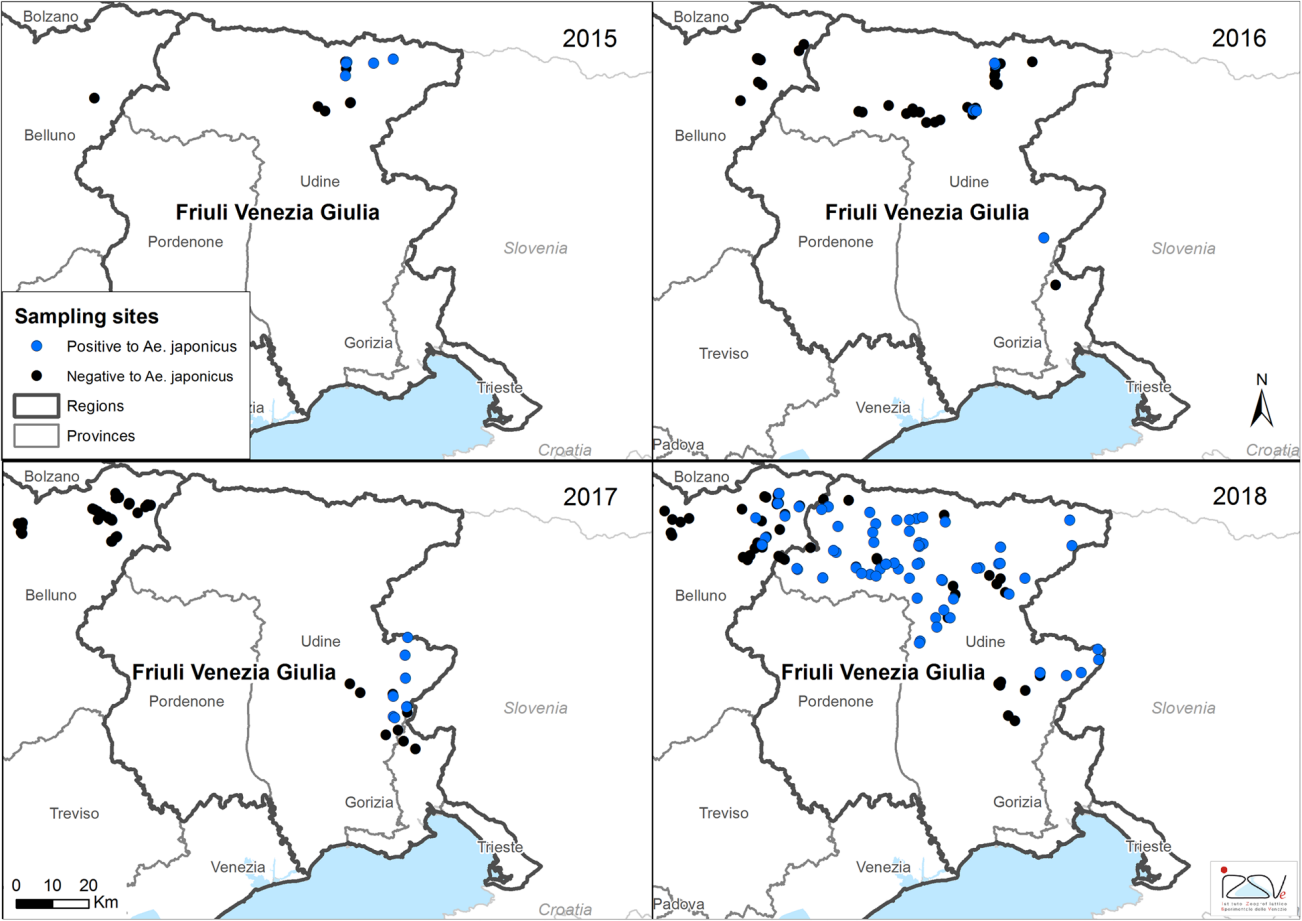
Map of sampling sites monitored for the presence of *Aedes j. japonicus* in northeastern Italy, 2015–2018. Black dots indicate sites negative for *Ae. j. japonicus* larvae; blue dots indicate sampling sites positive for *Ae. j. japonicus* larvae in 2015, 2016, 2017 and 2018, respectively

The current distribution of *Ae. j. japonicus* in northern Italy is reported in Fig. 1. To date, *Ae. j. japonicus* has been found in 58 municipalities out of 73 monitored (79.5%): 51/62 (82.3%) in FVG Region and 7/11 (63.6%) in Veneto Region (Table 1). The prevalence of positive municipalities in FVG Region increased every year, from 21.4% in 2016, to 41.7% in 2017 and 87.8% in 2018 (*χ*^2^ = 26.567, *df* = 2, *P* < 0.0001). In total (2015–2018), 238 sampling sites were monitored and 90 were positive for presence of *Ae. j. japonicus* larvae (37.8%) (Fig. 2, see Additional file 1: Table S1). In 2016, the species was found in one already positive municipality (Pontebba), in another one monitored but negative the previous year (Resiutta), and in a new site far away (Cividale del Friuli) by local authorities. In 2017, 5 out of 12 municipalities (41.7%), one of them already positive the previous year (Cividale del Friuli), and 8 out of 16 (50%) sampling sites were found infested. In 2018, most of the municipalities and sites monitored were positive to *Ae. j. japonicus* larvae (87.8 and 63.6% in FVG and Belluno Province, respectively) (Table 1). Notably, nine municipalities that were found positive in FVG in 2018 were negative in 2016. In Belluno Province, *Ae. j. japonicus* was found in a survey carried out in September 2018, while previously the same sites were negative. Notably, three positive municipalities in September were negative in samplings performed in May and July (see Additional file 1: Table S1). The mosquito spread from the valleys to the hilly and mountainous areas in the range of altitude between 99 masl. (Torreano, Province of Udine; 46°07′57″N, 13°25′56″E) and 1263 m a.s.l. (Sappada, Province of Udine; 46°34′13″N, 12°42′17.8″E).

**Table 1:** Municipalities, sampling sites and breeding sites monitored and positive for *Aedes j. japonicus* in northern Italy, 2015–2018

Year	Sampling sites	Breeding sites	Municipalities
	Positive/Monitored^a^ (%)	Positive/Monitored^b^ (%)	Positive/Monitored^a^ (%)
Friuli Venezia Giulia Region			
2015	5/12 (41.7)	5/12 (41.7)	2/5 (40.0)
2016	6/29 (20.7)	6/38 (15.8)	3/14 (21.4)
2017	8/16 (50.0)	8/21 (36.4)	5/12 (41.7)
2018	63/86 (73.3)	69/95 (71.9)	43/49 (87.8)
Veneto Region			
2015	0/3 (0)	0/4 (0)	0/1 (0)
2016	0/13 (0)	0/14 (0)	0/6 (0)
2017	0/37 (0)	0/40 (0)	0/7 (0)
2018	8/44 (18.2)	8/49 (16.3)	7/10 (63.6)

^a^Positive sites and municipalities each year may contain sites and municipalities positive the year before

^b^In each sampling site, multiple breeding sites may have been checked

*Aedes j. japonicus* larvae were found mainly in tires and in any kind of artificial containers, often located in private gardens (Table 2). In general, 52.2% of breeding sites checked, located in semi-urban areas (usually small villages), were positive to *Ae. j. japonicus* larvae. Compared to the other invasive mosquitoes occurring in the same area (*Ae. albopictus* and *Ae. koreicus*), *Ae. j. japonicus* was less present in catch basins and cemeteries, the last being positive only in two cases out of 17 monitored [14]. Over the period of sampling, the first larvae were observed in March 2016 and the last one in November 2018.

**Table 2:** Breeding sites monitored and positive for *Aedes j. japonicus* in Friuli Venezia Giulia Region (FVG) and Veneto Region (Belluno Province), 2015–2018

Breeding sites	FVG	Belluno	Total
	Positive/Monitored (%)	Positive/Monitored (%)	Positive/Monitored (%)
Tires	15/24 (62.5)	1/3 (33.3)	16/27 (59.3)
Big water containers	28/47 (59.6)	5/18 (27.8)	33/65 (50.8)
Small water containers	17/32 (53.1)	5/22 (22.7)	22/54 (40.7)
Vases/soucers	16/36 (44.4)	–	16/36 (44.4)
Catch basins	6/18 (33.3)	–	6/18 (33.3)
Basin of fountains	2/5 (40.0)	0/1 (0)	2/6 (33.3)
Puddles	0/1 (0)	0/1 (0)	0/2 (0)
Dunghills	0/1 (0)	0/1 (0)	0/2 (0)
Total	84/164 (51.2)	11/43 (25.6)	95/210 (45.2)

**Fig. 3:**
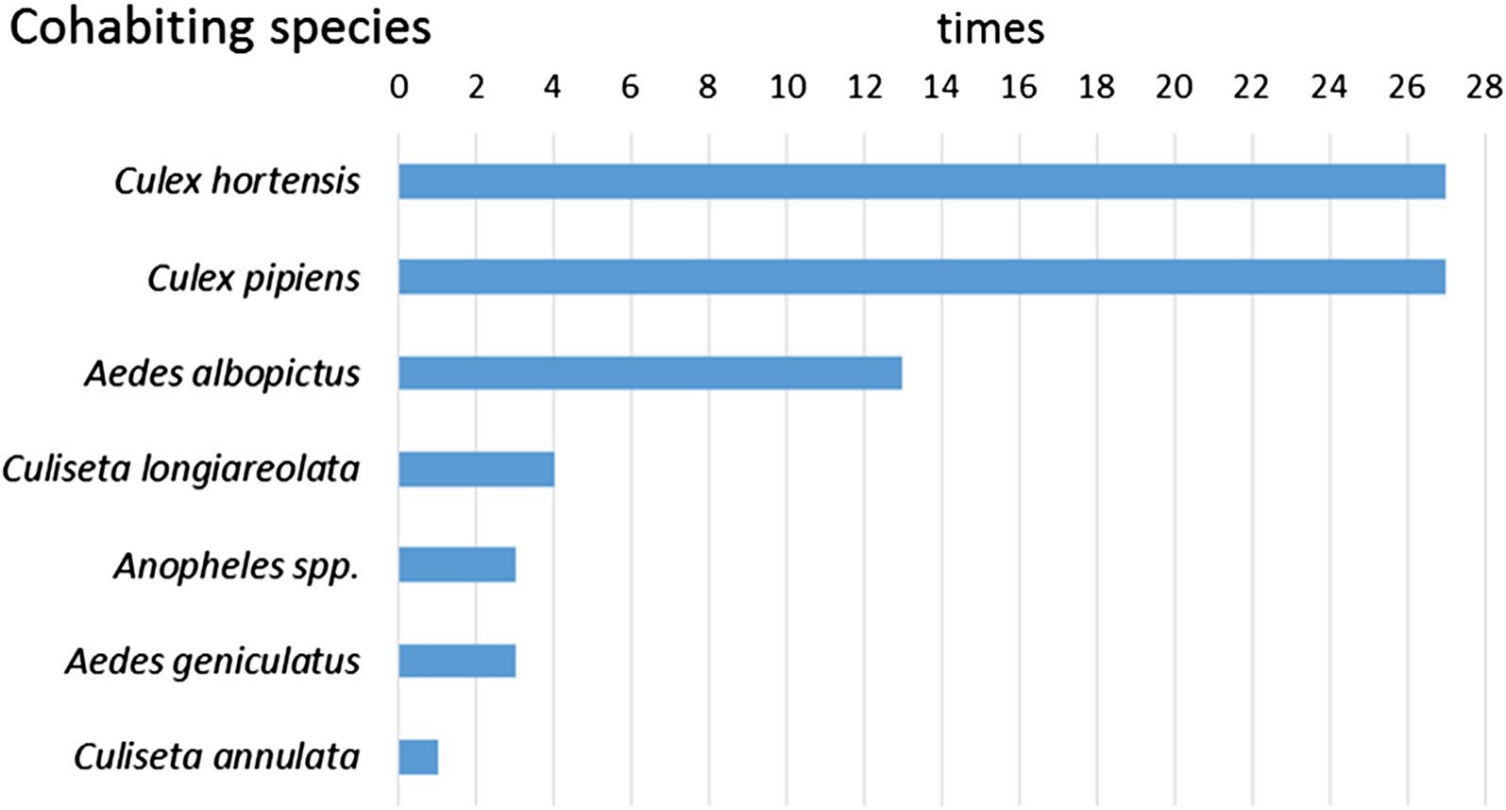
Number of times of recorded coexistence among *Aedes j. japonicus* larvae and other species

**Fig. 4:**
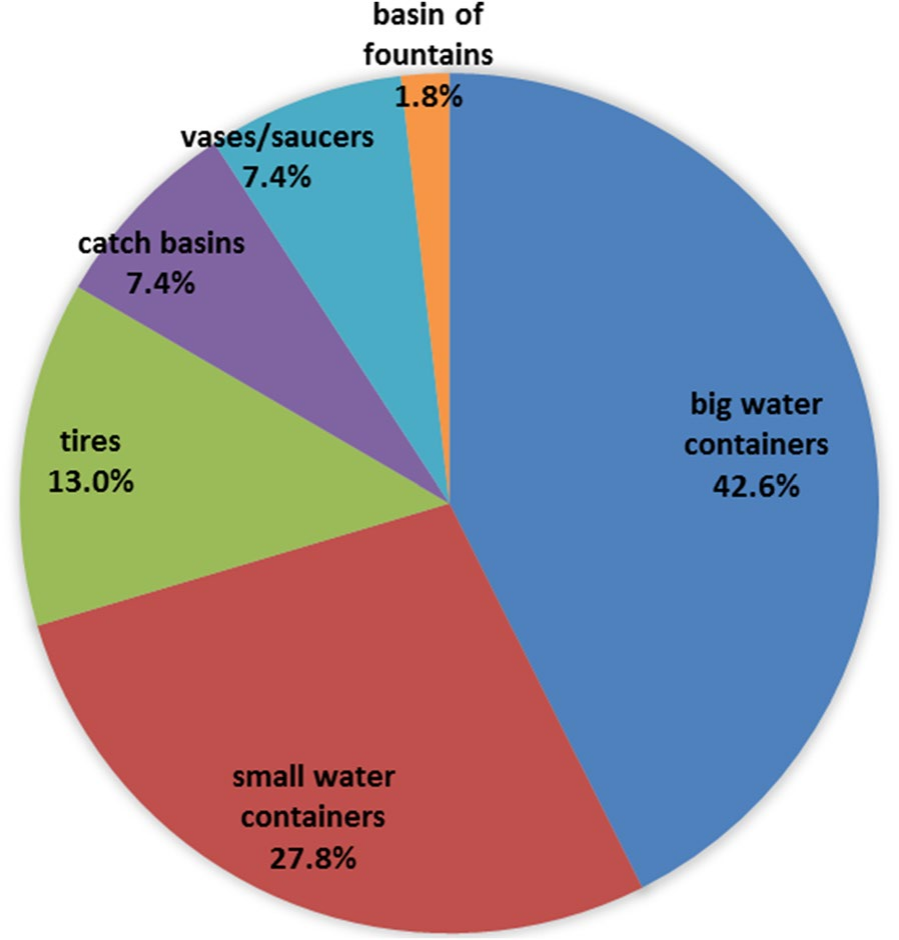
Percentage of breeding containers where co-existence among *Aedes j. japonicus* and other species was observed

During the survey, other mosquito larvae were collected belonging to 11 species: *Culex pipiens*, *Cx. hortensis*, *Anopheles maculipennis* (*s.l.*), *An. plumbeus*, *An. claviger/petragnani*, *Culiseta longiareolata*, *Cs. annulata*, *Aedes albopictus*, *Oc. geniculatus*, *Ae. koreicus* and *Oc. communis*. Cohabitation with other mosquito species was observed in 55.6% of the positive larval samplings. *Aedes j. japonicus* was associated with *Cx. hortensis* (27 times), *Cx. pipiens* (27 times) and with *Ae. albopictus* (13 times) (Fig. 3). Interestingly, *Ae. j. japonicus* was never found to share breeding sites with *Ae. koreicus*, even if their distribution partially overlapped (Fig. 1). Coexistence with at least one species was observed 29 times (58.0%), with another two species 15 times (30.0%), with three species twice (10.0%) and with four species once (2.0%). The breeding sites shared by *Ae. j. japonicus* and other mosquito larvae were mainly large and small artificial containers (Fig. 4). Large water containers were significantly more positive to cohabitation (42.6%) than tires (13.0%) (*χ*^2^ = 11.815, *df* = 1, *P* = 0.0006), catch basins and vases/saucers (7.4%) (*χ*^2^ = 17.827, *df* = 1, *P* < 0.0001) and basins of fountains (1.8%) (*χ*^2^ = 25.929, *df* = 1, *P* < 0.0001).

In total, 83 samples were submitted to PCR and 50 larvae and 11 adults were confirmed as *Ae. j. japonicus* with the three genes and with a similarity to GenBank sequences ranging between 98–100%. Other mosquitoes identified by molecular analysis were *Ae. koreicus* (*n* = 17), *Oc. communis* (*n* = 3), *Ae. albopictus* (*n* = 1) and *Cx. hortensis* (*n* = 1). Sequences of *Ae. j. japonicus* obtained by the three genes were submitted to the GenBank database under the accession numbers MK265679-MK265696.

## Discussion

After the first finding of *Ae. japonicus* in Italy in 2015 [10] this invasive mosquito spread throughout north Italy in the Provinces of Udine, bordering with Austria and Slovenia, and in the Province of Belluno and it is currently established in an area of approximately 3273 km^2^.

During 2016, the spread of *Ae. j. japonicus* seemed limited because it was absent around the municipalities positive in 2015 except for some collections in a municipality (Resiutta) 20 km to the southwest. In 2018, *Ae. j. japonicus* spread far, 40 km towards the west around the Alps and Alpine foothills infesting sites in FVG which were negative in 2016 and reaching the Belluno Province in September. In this part of Italy (Belluno Province) a well-organized IMS surveillance programme is ongoing and targeted to determine the distribution of *Ae. koreicus* [12] with frequent larval surveys; the species had not been found previously. Conversely, the spread towards the south was limited and the species did not reach the plain area of FVG Region. The rapid colonization observed in Italy is faster than in other European countries [7, 20, 21], suggesting environmental conditions particularly favorable to the development of this species. Indeed, it has been reported that in suitable habitats *Ae. j. japonicus* is able to increase its population within three years after the initial colonization [1, 7, 8, 22, 23].

The spread of *Ae. j. japonicus* for long distances is likely due to the transportation of eggs, larvae and adults by human activities through vehicles, while spread in close municipalities is due to the active expansion of the locally established population [1].

*Aedes j. japonicus* is a mosquito species adapted to tolerate cold temperatures. In a recent study based on predictive models of potential distribution species [24], the southernmost limits of this species in Europe have been indicated as within “a small region in northern Italy”, perfectly matching the area currently colonised. The expansion southwards seems to be limited by high mean temperatures (the average of mean temperature is over 32 °C for the warmest quarter in the plain area of the Region, [25]) and by the high density of the competitor species *Ae. albopictus* in the plain area [26, 27].

*Aedes j. japonicus* is confirmed to utilize artificial containers as main breeding sites [1, 27] and to be more common in natural and rural areas than in urban sites [28]. The capacity to establish in early spring and to be active until autumn is characteristic of this species, which is able to tolerate cold temperature [26]. Compared to other invasive mosquito species, the seasonal activity period is longer, lasting at least seven months (April-October); in our monitoring the first larvae were found on 29 March 2016 and on 26 April 2018 and the last on 12 October 2016 and on 08 November 2018. *Aedes j. japonicus* larvae pre-empted the finding of *Ae. albopictus* larvae of two months and remained active at least one more month, therefore decreasing the possibility of larval competition [29]. This phenology seems to require several generations per year, which occurs in areas with certain climatic characteristics, such as the winter not being extremely cold [26].

The ability of an invasive mosquito to establish in a new area is not only dependent on climate but also on the availability of empty ecological niches [27]. In our study, *Ae. j. japonicus* larvae were found mainly in areas too cold for *Ae. albopictus*, which occurs in approximately one-third of the area invaded by *Ae. j. japonicus* (Fig. 1).

Larval coexistence with other species was observed with other container-breeding mosquitoes. *Aedes j. japonicus* seemed not negatively affected by the presence of *Culex* spp. species, as reported elsewhere [30, 31] and multiple larval cohabitation was also possible, contrasting with reports of *Ae. j. japonicus* displacing native mosquitoes [20, 32, 33].

The rapid expansion of a new invasive mosquito and potential vector of pathogens may pose new threats to animals and humans. The vectorial role of *Ae. j. japonicus* has been assessed in laboratory studies which need confirmation in the field [1]. A potential vector competence for several viruses and nematodes (*Dirofilaria immitis* and *D. repens*) of medical and veterinary relevance has been suggested [10, 34, 35]. Notably, recent studies report that populations of *Ae. j. japonicus* collected in Switzerland are susceptible to West Nile virus (WNV) lineage 2 [36, 37]. As this mosquito species is an opportunistic feeder on mammals and birds [38] it could act as a bridge vector of WNV in Europe in case of considerable abundance. In the same geographical area of FVG Region colonised by *Ae. j. japonicus*, a high prevalence of *D. immitis* in stray dogs was reported [39], as well as circulation of WNV [40] in the lowland, an area not yet overlapping with the area colonised by *Ae. j. japonicus.* In the case of further spreading of *Ae. j. japonicus*, the risk of exposure to *D. immitis* and WNV could increase both for animals and humans, within areas previously considered at negligible risk in Italy, especially at high altitudes.

## Conclusions

This study shows that *Ae. j. japonicus* is well established in Italy and in only four years has rapidly colonised two Italian Regions throughout hilly and mountainous areas. According to these findings, northern Italy has a high probability of being invaded by *Ae. j. japonicus* in the future, possibly limited towards south by climatic conditions and occurrence of the larval competitor *Ae. albopictus*. The establishment of *Ae. j. japonicus* in an area where other invasive species occur has complicated the current entomological monitoring system, due to the similar biology and morphology. Therefore, a long-term surveillance and an early detection are needed to limit the further spread and plan control actions against this invasive mosquito.

## Additional file

**Additional file 1: Table S1.** Data, georeferenced position and description of sampling sites for *Ae. j. japonicus* surveillance.

## Abbreviations

FVG: Friuli Venezia Giulia
IMS: invasive mosquito species
*nad*4: nicotinamide adenine dinucleotide dehydrogenase subunit 4
*cox*1: cytochrome *c* oxidase subunit 1
BTUB: β tubulin
masl: meters above sea level
WNV: West Nile virus
PCR: polymerase chain reaction
