# Supplementary material for: The invasive mosquito Aedes japonicus japonicus is spreading in northeastern Italy
Source: Parasit Vectors. 2019 Mar 26;12:120. doi: 10.1186/s13071-019-3387-x (PMC6434805; doi:10.1186/s13071-019-3387-x)
Supplement: Supplementary file 1 — Additional file 1: Table S1. Data, georeferenced position and description of sampling sites for Ae. j. japonicus surveillance. [file 13071_2019_3387_MOESM1_ESM.docx]

**Additional file 1: Table S1.** Data, georeferenced position and description of sampling sites for *Ae. j. japonicus* surveillance

| **Data** | **Region** | **Municipality** | **Waypoint N** | **Waypoint E** | **Elevation (masl)** | **Site** | ***Aedes japonicus*** | ***Ae. albopctus*** | ***Ae. koreicus*** | ***Cx. pipiens*** | ***Cx. hortensis*** | ***Oc. geniculatus*** | ***Cs. longiareolata*** | ***Anopheles* spp.** | **Others** |
| --- | --- | --- | --- | --- | --- | --- | --- | --- | --- | --- | --- | --- | --- | --- | --- |
| 07/31/15 | FVG | Malborghetto Valbruna | 45,510494 | 13,470631 | 762 | private garden | X |  |  |  |  |  |  |  |  |
| 07/31/15 | FVG | Malborghetto Valbruna | 46,503130 | 13,400003 | 689 | private garden | X |  |  | X | X | X |  |  |  |
| 09/07/15 | FVG | Moggio Udinese | 46,403025 | 13,194072 | 312 | tire market |  | X |  | X |  |  |  |  |  |
| 09/07/15 | FVG | Resiutta | 46,391061 | 13,218086 | 326 | cemetery |  | X |  |  | X |  |  |  |  |
| 09/07/15 | FVG | Pontebba | 46,509566 | 13,299638 | 607 | private garden |  |  |  | X |  |  |  |  |  |
| 09/07/15 | FVG | Pontebba | 46,476144 | 13,297066 | 500 | private garden | X |  |  |  | X |  |  |  |  |
| 09/07/15 | FVG | Pontebba | 46,509744 | 13,304950 | 592 | private garden | X |  |  | X |  |  | X |  |  |
| 09/07/15 | FVG | Pontebba | 46,493304 | 13,300622 | 550 | cemetery |  |  |  |  |  |  |  |  |  |
| 09/07/15 | FVG | Pontebba | 46,507377 | 13,302130 | 569 | public park |  |  |  | X |  |  |  |  |  |
| 09/07/15 | FVG | Pontebba | 46,507244 | 13,302788 | 570 | park | X |  |  | X |  |  |  |  |  |
| 09/07/15 | FVG | Chiusaforte | 46,408463 | 13,308391 | 417 | cemetery |  |  |  |  |  |  |  |  |  |
| 09/07/15 | FVG | Chiusaforte | 46,409308 | 13,310886 | 392 | private garden |  |  |  |  |  |  |  |  |  |
| 08/05/15 | Veneto | Domegge di Cadore | 46,450598 | 12,398795 | 727 | private garden |  |  | X | X | X |  |  |  |  |
| 03/29/16 | FVG | Pontebba | 46,507378 | 13,302131 | 569 | public garden |  |  |  |  |  |  |  |  |  |
| 03/29/16 | FVG | Pontebba | 46,507244 | 13,302789 | 570 | park |  |  |  |  |  |  |  |  |  |
| 03/29/16 | FVG | Resiutta | 46,394701 | 13,213873 | 325 | private garden | X |  |  |  |  |  |  |  |  |
| 03/29/16 | FVG | Amaro | 46,375547 | 13,093430 | 417 | street |  |  |  |  |  |  |  |  |  |
| 06/28/16 | FVG | Cormons | 45,950989 | 13,473964 | 50 | garden center |  |  |  |  |  |  |  |  |  |
| 07/12/16 | FVG | Socchieve | 46,406293 | 12,804266 | 524 | private garden |  |  |  | X |  |  |  | X |  |
| 07/12/16 | FVG | Socchieve | 46,404234 | 12,816732 | 451 | private garden |  |  |  | X | X |  |  | X |  |
| 07/12/16 | FVG | Amaro | 46,370089 | 13,074802 | 274 | private garden |  | X |  | X |  |  |  | X |  |
| 07/12/16 | FVG | Moggio Udinese | 46,403025 | 13,194072 | 312 | tire market |  | X |  | X | X |  | X |  |  |
| 07/12/16 | FVG | Resiutta | 46,394185 | 13,224546 | 324 | farm | X | X |  | X | X |  |  |  |  |
| 07/12/16 | FVG | Resiutta | 46,393578 | 13,224617 | 325 | private garden | X | X |  | X |  |  |  |  |  |
| 07/12/16 | FVG | Chiusaforte | 46,461209 | 13,296371 | 458 | forest |  |  |  | X |  | X |  |  |  |
| 07/12/16 | FVG | Pontebba | 46,476144 | 13,297067 | 500 | private garden |  |  |  | X |  |  |  | X |  |
| 07/12/16 | FVG | Pontebba | 46,505139 | 13,302262 | 581 | private garden | X |  |  |  | X |  |  |  |  |
| 07/12/16 | FVG | Pontebba | 46,509566 | 13,299638 | 607 | private garden | X |  |  | X | X |  | X | X |  |
| 07/12/16 | FVG | Dogna | 46,455863 | 13,305736 | 452 | cemetery |  |  |  | X |  |  |  |  |  |
| 10/06/16 | FVG | Cividale del Friuli | 46,070149 | 13,441239 | 120 | street | X |  |  |  |  |  |  |  |  |
| 10/12/16 | FVG | Resiutta | 46,384713 | 13,211830 | 401 | street |  |  |  |  |  |  |  |  |  |
| 10/12/16 | FVG | Moggio Udinese | 46,399899 | 13,223483 | 394 | garden center |  |  |  |  |  |  |  |  |  |
| 10/12/16 | FVG | Pontebba | 46,506690 | 13,320180 | 579 | street |  |  |  | X | X |  |  |  |  |
| 10/12/16 | FVG | Pontebba | 46,479735 | 13,298953 | 506 | street |  |  |  |  |  |  |  |  |  |
| 10/12/16 | FVG | Pontebba | 46,493500 | 13,300530 | 599 | cemetery |  |  |  | X |  |  |  |  |  |
| 10/12/16 | FVG | Malborghetto Valbruna | 46,506937 | 13,434562 | 716 | cemetery |  |  |  | X |  |  |  |  |  |
| 10/12/16 | FVG | Cavazzo Carnico | 46,370354 | 13,044775 | 277 | private garden |  |  |  | X |  |  |  |  |  |
| 10/12/16 | FVG | Tolmezzo | 46,397253 | 13,022242 | 305 | street |  | X |  | X | X |  |  |  |  |
| 10/12/16 | FVG | Tolmezzo | 46,406460 | 12,998060 | 317 | street |  |  |  | X | X |  |  |  |  |
| 10/12/16 | FVG | Villa Santina | 46,417130 | 12,912070 | 373 | street |  |  |  | X |  |  |  |  |  |
| 10/12/16 | FVG | Verzegnis | 46,397914 | 13,000670 | 310 | street |  |  |  |  |  |  |  |  |  |
| 10/12/16 | FVG | Verzegnis | 46,395190 | 12,974540 | 437 | street |  |  |  | X |  |  | X |  | Cs. annulata |
| 07/13/16 | Veneto | Calalzo di Cadore | 46,446600 | 12,381986 | 799 | private garden |  |  | X | X |  |  |  |  |  |
| 07/13/16 | Veneto | Lozzo di Cadore | 46,490074 | 12,448675 | 774 | private garden |  |  |  | X |  |  |  |  |  |
| 07/13/16 | Veneto | Lorenzago di Cadore | 46,483587 | 12,46155 | 849 | cemetery |  |  |  | X |  |  |  |  |  |
| 07/13/16 | Veneto | Auronzo di Cadore | 46,545281 | 12,460037 | 867 | cemetery |  |  |  |  |  |  |  |  |  |
| 09/07/16 | Veneto | Auronzo di Cadore | 46,547678 | 12,451207 | 855 | depot |  |  |  | X |  |  |  |  |  |
| 09/07/16 | Veneto | Auronzo di Cadore | 46,546278 | 12,455845 | 857 | private garden |  |  |  |  | X |  |  |  |  |
| 09/07/16 | Veneto | Auronzo di Cadore | 46,54785 | 12,454279 | 872 | street |  |  |  |  |  |  |  |  | Culex sp. |
| 09/07/16 | Veneto | Auronzo di Cadore | 46,549094 | 12,448401 | 863 | farm |  |  |  |  |  |  |  |  |  |
| 09/07/16 | Veneto | Auronzo di Cadore | 46,548722 | 12,451024 | 861 | gas station |  |  |  |  |  |  |  |  |  |
| 09/07/16 | Veneto | Auronzo di Cadore | 46,547098 | 12,451808 | 850 | private garden |  |  |  |  |  |  |  |  |  |
| 09/07/16 | Veneto | Auronzo di Cadore | 46,547105 | 12,452590 | 854 | street |  |  |  |  |  |  |  |  |  |
| 09/07/16 | Veneto | Santo Stefano di Cadore | 46,563446 | 12,599773 | 943 | depot |  |  |  | X |  | X |  |  |  |
| 09/07/16 | Veneto | San Pietro di Cadore | 46,578904 | 12,618041 | 1008 | street |  |  |  |  | X |  |  |  |  |
| 06/21/17 | FVG | Povoletto | 46,109342 | 13,281788 | 130 | private garden |  | X |  | X |  |  |  |  |  |
| 06/21/17 | FVG | Remanzacco | 46,086572 | 13,317423 | 111 | street |  |  |  | X |  |  |  |  |  |
| 06/21/17 | FVG | Cividale del Friuli | 46,078694 | 13,431941 | 131 | cemetery |  | X |  |  |  |  |  |  |  |
| 06/21/17 | FVG | Cividale del Friuli | 46,073256 | 13,433454 | 127 | private garden | X |  |  |  |  |  |  |  |  |
| 06/21/17 | FVG | San Pietro Natisone | 46,116466 | 13,480509 | 151 | depot | X | X |  | X | X |  |  |  |  |
| 06/21/17 | FVG | Pulfero | 46,173624 | 13,483615 | 187 | private garden | X |  |  |  |  |  |  |  |  |
| 06/21/17 | FVG | Pulfero | 46,217035 | 13,496687 | 234 | depot | X |  |  |  |  |  |  |  |  |
| 07/26/17 | FVG | Cormons | 45,960774 | 13,460629 | 63 | street |  | X |  | X |  |  |  |  |  |
| 07/26/17 | FVG | Capriva del Friuli | 45,939526 | 13,501694 | 45 | garden center |  | X |  | X |  |  |  | X |  |
| 07/26/17 | FVG | Corno di Rosazzo | 45,989289 | 13,444043 | 80 | private garden |  | X |  |  |  |  |  |  |  |
| 07/26/17 | FVG | San Giovanni al Natisone | 45,978979 | 13,399417 | 65 | street |  | X |  | X |  |  |  |  |  |
| 07/26/17 | FVG | Dolegna | 46,031522 | 13,479881 | 86 | square |  | X |  |  |  |  |  |  |  |
| 07/26/17 | FVG | Prepotto | 46,045778 | 13,479439 | 108 | street | X |  |  |  |  |  |  |  |  |
| 07/26/17 | FVG | Prepotto | 46,045669 | 13,478005 | 103 | private garden | X | X |  | X |  |  |  |  |  |
| 09/22/17 | FVG | Premariacco | 46,023756 | 13,429596 | 168 | private garden | X |  |  |  |  |  |  |  |  |
| 09/22/17 | FVG | Premariacco | 46,020622 | 13,433720 | 150 | private garden | X |  |  |  |  |  |  |  |  |
| 07/05/17 | Veneto | Auronzo di Cadore | 46,558273 | 12,427357 | 876 | private garden |  |  |  |  | X |  |  |  |  |
| 07/05/17 | Veneto | Auronzo di Cadore | 46,547675 | 12,451205 | 856 | private garden |  |  |  |  | X |  |  |  |  |
| 07/05/17 | Veneto | Auronzo di Cadore | 46,566061 | 12,412412 | 895 | depot |  |  | X |  |  |  |  |  |  |
| 07/05/17 | Veneto | Auronzo di Cadore | 46,542174 | 12,462503 | 845 | private garden |  |  |  | X |  |  |  |  |  |
| 07/05/17 | Veneto | Auronzo di Cadore | 46,571282 | 12,400817 | 918 | private garden |  |  |  | X |  |  |  |  | Culex sp. |
| 07/05/17 | Veneto | Auronzo di Cadore | 46,573369 | 12,394168 | 914 | depot |  |  |  |  |  |  |  |  |  |
| 07/05/17 | Veneto | Auronzo di Cadore | 46,54251 | 12,462662 | 853 | private garden |  |  |  |  |  |  |  |  |  |
| 07/05/17 | Veneto | Auronzo di Cadore | 46,54513 | 12,460310 | 868 | cemetery |  |  |  |  |  |  |  |  |  |
| 07/05/17 | Veneto | Auronzo di Cadore | 46,551255 | 12,445916 | 883 | cemetery |  |  |  |  |  |  |  |  |  |
| 07/05/17 | Veneto | Cortina d'Ampezzo | 46,542147 | 12,128216 | 1223 | private garden |  |  |  |  | X |  |  |  |  |
| 07/06/17 | Veneto | Cortina d'Ampezzo | 46,545870 | 12,412837 | 1286 | depot |  |  |  |  | X |  |  |  | Culex sp. |
| 07/05/17 | Veneto | Cortina d'Ampezzo | 46,544445 | 12,126282 | 1260 | private garden |  |  |  |  | X |  |  |  |  |
| 07/06/17 | Veneto | Cortina d'Ampezzo | 46,534531 | 12,138325 | 1180 | cemetery |  |  |  | X | X |  |  |  |  |
| 07/06/17 | Veneto | Cortina d'Ampezzo | 46,544975 | 12,14224 | 1275 | private garden |  |  |  | X | X |  |  |  |  |
| 07/06/17 | Veneto | Cortina d'Ampezzo | 46,522233 | 12,135172 | 1126 | depot |  |  |  | X |  |  |  |  |  |
| 07/06/17 | Veneto | Cortina d'Ampezzo | 46,51892 | 12,139716 | 1159 | depot |  |  |  | X |  |  |  |  | Culex sp. |
| 07/26/17 | Veneto | Vigo di Cadore | 46,500025 | 12,474598 | 942 | private garden |  |  |  |  | X |  |  |  |  |
| 07/26/17 | Veneto | Vigo di Cadore | 46,500375 | 12,473336 | 938 | private garden |  |  |  | X | X |  |  |  |  |
| 07/26/17 | Veneto | Vigo di Cadore | 46,500343 | 12,474151 | 940 | private garden |  |  |  | X |  |  |  |  |  |
| 07/26/17 | Veneto | Vigo di Cadore | 46,501812 | 12,477682 | 901 | private garden |  |  |  |  | X |  |  |  |  |
| 07/26/17 | Veneto | Vigo di Cadore | 46,490136 | 12,459417 | 797 | private garden |  |  |  | X |  |  |  |  |  |
| 07/26/17 | Veneto | Vigo di Cadore | 46,490382 | 12,457784 | 782 | private garden |  |  | X |  |  |  |  |  |  |
| 07/26/17 | Veneto | Vigo di Cadore | 46,490034 | 12,458727 | 788 | private garden |  |  | X |  |  |  |  |  |  |
| 08/08/17 | Veneto | San Pietro di Cadore | 46,571739 | 12,584250 | 1036 | cemetery |  |  |  |  |  |  |  |  |  |
| 08/08/17 | Veneto | San Pietro di Cadore | 46,577126 | 12,592801 | 1170 | private garden |  |  |  |  | X |  |  |  | Cs. annulata |
| 08/08/17 | Veneto | San Pietro di Cadore | 46,576737 | 12,592685 | 1157 | private garden |  |  |  |  | X |  |  |  |  |
| 08/08/17 | Veneto | San Pietro di Cadore | 46,576175 | 12,603598 | 977 | private garden |  |  |  | X |  |  |  |  |  |
| 08/08/17 | Veneto | San Pietro di Cadore | 46,574918 | 12,602350 | 977 | depot |  |  |  | X | X |  |  | X |  |
| 08/08/17 | Veneto | San Nicolò di Comelico | 46,582503 | 12,526832 | 1057 | private garden |  |  |  | X | X |  |  |  |  |
| 08/08/17 | Veneto | San Nicolò di Comelico | 46,582321 | 12,527859 | 1065 | private garden |  |  |  | X |  |  |  |  |  |
| 08/08/17 | Veneto | San Nicolò di Comelico | 46,580490 | 12,525261 | 986 | private garden |  |  |  | X |  |  |  |  |  |
| 08/08/17 | Veneto | San Nicolò di Comelico | 46,580626 | 12,526193 | 993 | private garden |  |  |  | X |  |  |  |  |  |
| 08/08/17 | Veneto | Comelico Superiore | 46,596563 | 12,480181 | 1256 | depot |  |  |  |  | X |  |  |  | Culex sp. |
| 08/08/17 | Veneto | Comelico Superiore | 46,608700 | 12,479884 | 1201 | depot |  |  |  | X | X |  | X |  |  |
| 08/08/17 | Veneto | Comelico Superiore | 46,598458 | 12,490996 | 1221 | depot |  |  |  | X |  |  |  |  |  |
| 08/08/17 | Veneto | Comelico Superiore | 46,597564 | 12,490516 | 1197 | depot |  |  |  | X |  | X |  |  |  |
| 08/09/17 | Veneto | Santo Stefano di Cadore | 46,558401 | 12,555529 | 922 | depot |  |  |  | X | X |  |  |  |  |
| 04/26/18 | FVG | Tolmezzo | 46,419572 | 13,021728 | 354 | tire market | X |  |  | X |  | X |  |  |  |
| 04/26/18 | FVG | Tolmezzo | 46,417002 | 13,013686 | 350 | farm | X |  |  | X |  |  |  |  |  |
| 04/26/18 | FVG | Villa Santina | 46,40788 | 12,944694 | 349 | farm | X |  |  |  |  |  |  |  |  |
| 04/26/18 | FVG | Forni di Sopra | 46,418768 | 12,584154 | 887 | private garden | X |  |  |  |  |  |  |  |  |
| 04/26/18 | FVG | Forni di Sopra | 46,421211 | 12,581555 | 880 | public garden | X |  |  |  |  |  |  |  |  |
| 04/26/18 | FVG | Forni di Sopra | 46,444590 | 12,539244 | 1045 | private garden |  |  |  |  |  |  |  |  |  |
| 04/26/18 | FVG | Ampezzo | 46,415863 | 12,794282 | 552 | private garden | X |  |  |  |  |  |  |  |  |
| 04/26/18 | FVG | Ampezzo | 46,417296 | 12,793794 | 553 | private garden |  |  |  |  | X |  |  |  |  |
| 05/24/18 | FVG | Gemona del Friuli | 46,280215 | 13,122031 | 200 | tire market | X | X |  |  | X |  |  |  |  |
| 05/24/18 | FVG | Gemona del Friuli | 46,280454 | 13,113412 | 198 | private garden |  |  |  | X |  |  |  |  |  |
| 05/24/18 | FVG | Chiusaforte | 46,408006 | 13,305266 | 379 | private garden | X |  |  |  |  |  |  |  |  |
| 05/24/18 | FVG | Chiusaforte | 46,408301 | 13,310266 | 380 | square | X |  |  |  |  |  |  |  |  |
| 05/24/18 | FVG | Chiusaforte | 46,408836 | 13,311317 | 380 | cemetery | X |  |  |  |  |  |  |  |  |
| 05/24/18 | FVG | Resia | 46,379463 | 13,269767 | 362 | private garden |  |  |  |  |  |  |  |  |  |
| 05/24/18 | FVG | Resia | 46,369877 | 13,309037 | 450 | public garden |  |  |  |  |  |  |  |  |  |
| 05/24/18 | FVG | Resia | 46,357633 | 13,295024 | 547 | private garden |  |  |  |  |  |  |  |  |  |
| 05/24/18 | FVG | Resia | 46,334998 | 13,324269 | 1166 | private garden |  |  |  |  |  |  |  |  |  |
| 05/24/18 | FVG | Venzone | 46,358236 | 13,138009 | 250 | private garden |  |  |  |  | X |  |  |  |  |
| 05/24/18 | FVG | Venzone | 46,336028 | 13,144218 | 258 | private garden |  |  |  | X | X |  |  |  |  |
| 05/24/18 | FVG | Venzone | 46,326300 | 13,136710 | 225 | cemetery | X |  |  | X |  |  |  |  |  |
| 05/25/18 | FVG | Povoletto | 46,109444 | 13,282063 | 129 | farm |  |  |  | X |  |  |  |  |  |
| 05/25/18 | FVG | Povoletto | 46,105455 | 13,287009 | 127 | farm |  | X |  | X | X |  |  |  |  |
| 05/25/18 | FVG | Povoletto | 46,114308 | 13,291082 | 131 | private garden |  | X |  | X |  |  |  |  |  |
| 05/25/18 | FVG | Pradamano | 46,02955 | 13,310619 | 83 | farm |  |  |  | X |  |  |  |  |  |
| 05/25/18 | FVG | Buttrio | 46,0155167 | 13,333333 | 84 | street |  | X |  |  |  |  |  | X |  |
| 05/25/18 | FVG | Trasaghis | 46,2825333 | 13,069849 | 196 | private garden | X | X |  | X | X |  |  |  |  |
| 05/25/18 | FVG | Forgaria | 46,226235 | 13,010632 | 183 | private garden | X |  |  |  | X |  |  |  |  |
| 05/25/18 | FVG | Forgaria | 46,221559 | 13,007683 | 183 | street | X | X |  | X |  |  |  |  |  |
| 05/25/18 | FVG | Cavazzo Carnico | 46,33333 | 13,0076683 | 215 | private garden | X |  |  |  |  |  |  |  |  |
| 05/25/18 | FVG | Verzegnis | 46,383333 | 12,983333 | 441 | tire market | X |  |  |  | X |  |  |  |  |
| 05/25/18 | FVG | Bordano | 46,300000 | 13,100000 | 226 | private garden | X |  |  | X | X |  |  |  |  |
| 06/26/18 | FVG | Sappada | 46,570281 | 12,704962 | 1263 | private garden | X |  |  | X |  |  |  |  |  |
| 06/26/18 | FVG | Sappada | 46,564924 | 12,680328 | 1214 | farm | X |  |  | X |  |  |  |  | Cs. annulata |
| 06/26/18 | FVG | Forni Avoltri | 46,584033 | 12,779403 | 864 | public garden |  |  |  | X |  |  |  |  |  |
| 07/13/18 | FVG | Amaro | 46,373859 | 13,101094 | 285 | private garden | X |  |  |  |  |  |  |  |  |
| 07/13/18 | FVG | Amaro | 46,375541 | 13,09785 | 311 | private garden | X |  |  |  |  |  |  |  |  |
| 07/13/18 | FVG | Enemonzo | 46,410323 | 12,880177 | 392 | private garden | X |  |  |  |  |  |  |  |  |
| 07/13/18 | FVG | Sauris | 46,456416 | 12,725149 | 1024 | street | X |  |  |  |  |  |  |  |  |
| 07/13/18 | FVG | Sauris | 46,46125 | 12,715596 | 1116 | private garden | X |  |  | X |  |  | X |  |  |
| 07/13/18 | FVG | Forni di Sotto | 46,395096 | 12,673446 | 783 | street | X |  |  |  |  |  |  |  |  |
| 07/13/18 | FVG | Forni di Sotto | 46,394817 | 12,67283 | 780 | private garden | X |  |  |  | X |  |  |  |  |
| 07/30/18 | FVG | Arta Terme | 46,468949 | 13,026500 | 429 | street | X |  |  | X |  |  |  |  |  |
| 07/30/18 | FVG | Arta Terme | 46,469944 | 13,026062 | 429 | street | X |  |  |  |  |  |  |  |  |
| 07/30/18 | FVG | Arta Terme | 46,467500 | 13,038611 | 429 | private garden | X |  |  |  | X |  |  |  |  |
| 07/30/18 | FVG | Zuglio | 46,461572 | 13,024361 | 424 | private garden | X |  |  |  |  |  |  |  |  |
| 07/30/18 | FVG | Zuglio | 46,463872 | 13,022913 | 426 | private garden | X |  |  |  |  |  |  |  |  |
| 07/30/18 | FVG | Lauco | 46,42351 | 12,931533 | 723 | private garden | X |  |  | X |  |  |  |  |  |
| 08/28/18 | FVG | Socchieve | 46,39728 | 12,843283 | 430 | street | X |  |  | X | X |  |  |  |  |
| 08/28/18 | FVG | Socchieve | 46,40168 | 12,813215 | 430 | private garden | X |  |  | X |  |  |  |  |  |
| 08/28/18 | FVG | Preone | 46,39327 | 12,864566 | 440 | private garden | X |  |  |  | X |  | X | X |  |
| 08/28/18 | FVG | Raveo | 46,433108 | 12,871583 | 450 | private garden |  |  |  | X | X |  |  |  |  |
| 09/06/18 | FVG | Osoppo | 46,258871 | 13,072637 | 200 | street | X |  |  |  |  |  |  |  |  |
| 09/20/18 | FVG | Resia | 46,303116 | 13,336712 | 580 | street | X |  |  |  |  |  |  |  |  |
| 09/20/18 | FVG | Resia | 46,306808 | 13,396822 | 575 | depot | X |  |  |  |  |  |  |  |  |
| 09/20/18 | FVG | Resia | 46,357489 | 13,295288 | 513 | private garden |  |  |  |  |  |  |  |  |  |
| 09/20/18 | FVG | Tarvisio | 46,505891 | 13,568332 | 748 | tire market | X |  |  |  |  |  |  |  |  |
| 09/20/18 | FVG | Tarvisio | 46,44213 | 13,571056 | 908 | private garden | X |  |  | X | X |  |  |  |  |
| 09/24/18 | FVG | San Leonardo | 46,121111 | 13,525278 | 138 | private garden | X | X |  |  | X |  |  |  |  |
| 09/25/18 | FVG | Stregna | 46,126667 | 13,577778 | 314 | private garden | X | X |  |  | X |  |  |  |  |
| 09/25/18 | FVG | Grimacco | 46,155833 | 13,644444 | 550 | private garden | X | X |  | X |  |  |  |  |  |
| 09/25/18 | FVG | Grimacco | 46,156389 | 13,643611 | 565 | private garden | X |  |  |  |  | X |  |  |  |
| 09/25/18 | FVG | Drenchia | 46,182500 | 13,642778 | 664 | private garden | X |  |  |  |  |  |  |  |  |
| 09/25/18 | FVG | Cividale del Friuli | 46,124444 | 13,431389 | 90 | private garden |  | X |  |  |  |  |  |  |  |
| 09/25/18 | FVG | Torreano | 46,132778 | 13,432500 | 105 | private garden | X | X |  |  |  |  |  |  |  |
| 09/25/18 | FVG | Torreano | 46,131667 | 13,432222 | 99 | private garden | X | X |  |  |  |  |  |  |  |
| 09/25/18 | FVG | Moimacco | 46,090000 | 13,375833 | 117 | private garden |  | X |  |  |  |  | X | X |  |
| 10/08/18 | FVG | Paluzza | 46,530656 | 13,019107 | 606 | private garden | X |  |  |  | X |  |  |  |  |
| 10/16/18 | FVG | Dogna | 46,448844 | 13,315887 | 431 | street | X |  |  |  |  |  |  |  |  |
| 10/16/18 | FVG | Dogna | 46,449052 | 13,315321 | 440 | public garden | X |  |  |  | X |  |  |  |  |
| 10/16/18 | FVG | Moggio Udinese | 46,400485 | 13,237648 | 413 | street | X |  |  |  |  |  |  | X |  |
| 10/16/18 | FVG | Moggio Udinese | 46,399221 | 13,226858 | 418 | private garden | X |  |  |  |  |  |  |  |  |
| 10/16/18 | FVG | Paularo | 46,518575 | 13,122169 | 625 | private garden | X | X |  |  |  |  |  |  |  |
| 10/16/18 | FVG | Treppo Ligosullo | 46,53505 | 13,120312 | 672 | forest |  |  |  |  |  |  |  |  |  |
| 10/16/18 | FVG | Treppo Ligosullo | 46,532971 | 13,042283 | 662 | private garden | X |  |  |  | X |  |  |  |  |
| 10/16/18 | FVG | Cercivento | 46,528833 | 12,993524 | 600 | street | X |  |  |  | X |  |  |  |  |
| 10/16/18 | FVG | Sutrio | 46,500289 | 12,991941 | 558 | farm | X |  |  |  |  |  |  |  |  |
| 10/16/18 | FVG | Ravascletto | 46,529257 | 12,948607 | 880 | street | X |  |  |  |  |  |  |  |  |
| 11/07/18 | FVG | Rigolato | 46,551875 | 12,852664 | 760 | private garden | X |  |  |  |  |  |  |  |  |
| 11/07/18 | FVG | Comeglians | 46,52291 | 12,871983 | 553 | private garden | X |  |  |  |  |  |  |  |  |
| 11/07/18 | FVG | Prato carnico | 46,520835 | 12,736404 | 713 | private garden | X |  |  |  | X |  |  |  |  |
| 11/07/18 | FVG | Ovaro | 46,502062 | 12,859742 | 525 | private garden | X |  |  | X |  |  |  |  |  |
| 11/07/18 | FVG | Raveo | 46,420534 | 12,901728 | 518 | private garden |  |  |  |  | X |  |  |  |  |
| 11/08/18 | FVG | Raveo | 46,420869 | 12,902922 | 399 | private garden | X |  |  |  |  |  |  |  |  |
| 11/08/18 | FVG | Raveo | 46,432991 | 12,870892 | 526 | private garden |  |  |  |  | X |  |  |  |  |
| 11/08/18 | FVG | Raveo | 46,436589 | 12,870427 | 526 | street |  |  |  |  | X |  |  |  |  |
| 11/08/18 | FVG | Ovaro | 46,476994 | 12,862746 | 488 | private garden | X |  |  |  | X |  |  |  |  |
| 04/26/18 | Veneto | Lorenzago di Cadore | 46,453543 | 12,518486 | 1289 | private garden |  |  |  |  |  |  |  |  |  |
| 04/26/18 | Veneto | Lorenzago di Cadore | 46,449204 | 12,528114 | 1112 | forest |  |  |  |  |  |  |  |  | Oc. communis |
| 05/02/18 | Veneto | Lozzo di Cadore | 46,474878 | 12,434620 | 798 | depot |  |  | X |  |  |  |  |  |  |
| 05/02/18 | Veneto | Lorenzago di Cadore | 46,483245 | 12,458118 | 851 | private garden |  |  |  |  | X |  |  |  |  |
| 05/02/18 | Veneto | Lorenzago di Cadore | 46,476986 | 12,461169 | 907 | private garden |  |  |  |  | X |  |  |  |  |
| 05/02/18 | Veneto | Lorenzago di Cadore | 46,476580 | 12,460110 | 889 | private garden |  |  |  |  |  |  |  |  |  |
| 07/25/18 | Veneto | Lorenzago di Cadore | 46,701944 | 12,634444 | 978 | public garden |  |  |  |  |  | X |  |  |  |
| 07/25/18 | Veneto | Lorenzago di Cadore | 46,588056 | 12,688333 | 1236 | public garden |  |  |  |  | X | X |  |  |  |
| 07/25/18 | Veneto | Vigo di Cadore | 46,518611 | 12,527500 | 942 | private garden |  |  | X |  | X |  |  |  | Culex sp. |
| 07/25/18 | Veneto | San Nicolò di Comelico | 46,683889 | 12,787500 | 968 | public garden |  |  |  |  | X |  |  |  |  |
| 08/22/18 | Veneto | Cortina d'Ampezzo | 46,515606 | 12,139455 | 1124 | private garden |  |  |  |  | X |  | X |  |  |
| 08/22/18 | Veneto | Cortina d'Ampezzo | 46,522274 | 12,135354 | 1124 | depot |  |  |  |  | X |  |  | X |  |
| 08/22/18 | Veneto | Cortina d'Ampezzo | 46,576687 | 12,116167 | 1298 | tire market |  |  |  | X |  |  |  |  |  |
| 08/22/18 | Veneto | Cortina d'Ampezzo | 46,556354 | 12,202306 | 1807 | street |  |  |  |  |  |  |  |  |  |
| 08/22/18 | Veneto | Cortina d'Ampezzo | 46,548260 | 12,171664 | 1588 | depot |  |  |  |  |  |  |  |  |  |
| 08/22/18 | Veneto | Cortina d'Ampezzo | 46,554991 | 12,143213 | 1411 | private garden |  |  |  |  |  |  |  |  |  |
| 09/10/18 | Veneto | Auronzo di Cadore | 46,573300 | 12,394200 | 914 | depot |  |  |  | X |  |  |  |  |  |
| 09/10/18 | Veneto | Auronzo di Cadore | 46,542600 | 12,462800 | 858 | private garden |  |  |  |  | X |  |  |  |  |
| 09/10/18 | Veneto | Auronzo di Cadore | 46,551033 | 12,442174 | 856 | private garden | X |  |  |  | X |  |  |  |  |
| 09/10/18 | Veneto | Santo Stefano di Cadore | 46,552000 | 12,548000 | 915 | depot | X |  |  | X |  |  |  |  |  |
| 09/10/18 | Veneto | Santo Stefano di Cadore | 46,556200 | 12,548000 | 909 | depot |  |  |  |  | X |  |  |  |  |
| 09/10/18 | Veneto | San Pietro di Cadore | 46,575200 | 12,602700 | 976 | street |  |  |  |  | X |  |  |  |  |
| 09/10/18 | Veneto | San Pietro di Cadore | 46,573400 | 12,600000 | 965 | depot | X |  |  |  |  |  |  |  |  |
| 09/10/18 | Veneto | Vigo di Cadore | 46,500100 | 12,474600 | 942 | private garden |  |  |  |  | X |  |  |  |  |
| 09/10/18 | Veneto | Vigo di Cadore | 46,500137 | 12,474203 | 940 | depot | X |  |  | X |  |  |  |  |  |
| 09/10/18 | Veneto | Vigo di Cadore | 46,501800 | 12,477800 | 901 | depot |  |  | X |  | X |  |  |  |  |
| 09/10/18 | Veneto | Lorenzago di Cadore | 46,484700 | 12,461800 | 823 | depot | X |  |  |  |  |  |  |  |  |
| 09/10/18 | Veneto | Lorenzago di Cadore | 46,483500 | 12,461000 | 846 | cemetery | X |  |  | X | X |  |  |  |  |
| 09/10/18 | Veneto | Domegge di Cadore | 46,453800 | 12,391700 | 914 | private garden |  |  |  |  |  |  |  |  |  |
| 09/10/18 | Veneto | Domegge di Cadore | 46,457600 | 12,416000 | 742 | depot |  |  | X |  | X |  |  |  |  |
| 09/10/18 | Veneto | Domegge di Cadore | 46,457155 | 12,414481 | 741 | private garden |  |  | X |  |  |  |  |  |  |
| 09/10/18 | Veneto | Domegge di Cadore | 46,447367 | 12,406220 | 685 | public garden |  |  | X |  |  |  |  |  |  |
| 09/25/18 | Veneto | Lozzo di Cadore | 46,483857 | 12,444772 | 743 | private garden |  |  | X | X |  |  |  |  |  |
| 09/26/18 | Veneto | San Nicolò di Comelico | 46,584667 | 12,524670 | 1056 | depot | X |  |  |  | X |  |  |  |  |
| 09/25/18 | Veneto | San Nicolò di Comelico | 46,582727 | 12,520062 | 1002 | depot |  |  |  | X | X |  |  |  |  |
| 09/25/18 | Veneto | Comelico Superiore | 46,609282 | 12,529747 | 1178 | depot | X |  |  |  |  |  |  |  |  |
| 09/25/18 | Veneto | Comelico Superiore | 46,605026 | 12,525364 | 1137 | depot |  |  |  | X |  |  |  |  |  |
| 09/25/18 | Veneto | Comelico Superiore | 46,600301 | 12,483126 | 1203 | depot |  |  |  | X |  |  |  |  |  |
| 09/25/18 | Veneto | Lozzo di Cadore | 46,487493 | 12,445055 | 787 | private garden |  |  |  | X |  |  |  |  |  |
| 09/25/18 | Veneto | Lozzo di Cadore | 46,488000 | 12,445200 | 788 | depot |  |  | X |  | X |  |  |  |  |
| 09/25/18 | Veneto | Comelico Superiore | 46,587700 | 12,524100 | 1188 | cemetery |  |  |  |  |  |  |  |  |  |
| 09/25/18 | Veneto | San Nicolò di Comelico | 46,608945 | 12,528909 | 1180 | depot |  |  |  |  |  |  |  |  |  |
| 09/25/18 | Veneto | Comelico Superiore | 46,602576 | 12,478175 | 1221 | private garden |  |  |  |  |  |  |  |  |  |
| 09/26/18 | Veneto | Lozzo di Cadore | 46,474950 | 12,434637 | 799 | private garden |  |  |  |  |  |  |  |  |  |
| FVG= Friuli Venezia Giulia | |  |  |  |  |  |  |  |  |  |  |  |  |  |  |
